# Effects of the Dual FAAH/MAGL Inhibitor AKU-005 on Trigeminal Hyperalgesia in Male Rats

**DOI:** 10.3390/cells13100830

**Published:** 2024-05-13

**Authors:** Rosaria Greco, Chiara Demartini, Miriam Francavilla, Anna Maria Zanaboni, Sara Facchetti, Michela Palmisani, Valentina Franco, Cristina Tassorelli

**Affiliations:** 1 Section of Translational Neurovascular Research, IRCCS Mondino Foundation, Via Mondino 2, 27100 Pavia, Italy; chiara.demartini@mondino.it (C.D.); miriam.francavilla@mondino.it (M.F.); annamaria.zanaboni@unipv.it (A.M.Z.); sara.facchetti@mondino.it (S.F.); michela.palmisani@mondino.it (M.P.); valentina.franco@unipv.it (V.F.); cristina.tassorelli@unipv.it (C.T.); 2 Department of Brain and Behavioral Sciences, University of Pavia, Via Bassi 21, 27100 Pavia, Italy; 3 Department of Internal Medicine and Therapeutics, Clinical and Experimental Pharmacology Unit, University of Pavia, Viale Golgi 19, 27100 Pavia, Italy

**Keywords:** endocannabinoids, FAAH, MAGL, migraine, pain

## Abstract

The inhibition of endocannabinoid hydrolysis by enzymatic inhibitors may interfere with mechanisms underlying migraine-related pain. The dual FAAH/MAGL inhibitor AKU-005 shows potent inhibitory activity in vitro. Here, we assessed the effect of AKU-005 in a migraine animal model based on nitroglycerin (NTG) administration. Male rats were treated with AKU-005 (0.5 mg/kg, i.p.) or vehicle 3 h after receiving NTG (10 mg/kg, i.p.) or NTG vehicle. One hour later, rats were subjected to the open field test followed by the orofacial formalin test. At the end of the test, we collected serum samples for assessing calcitonin gene-related peptide (CGRP) levels as well as meninges, trigeminal ganglia, and brain areas to assess mRNA levels of CGRP and pro-inflammatory cytokines, and endocannabinoid and related lipid levels. AKU-005 reduced NTG-induced hyperalgesia during the orofacial formalin test but did not influence NTG-induced changes in the open field test. It significantly reduced serum levels of CGRP, CGRP, and pro-inflammatory cytokine mRNA levels in the meninges, trigeminal ganglia, and central areas. Surprisingly, AKU-005 caused no change in endocannabinoids and related lipids in the regions evaluated. The present findings suggest that AKU-005 may have anti-migraine effects by reducing CGRP synthesis and release and the associated inflammatory events. This effect, however, does not seem mediated via an interference with the endocannabinoid pathway.

## 1. Introduction

The endocannabinoids N-arachidonoylethanolamine (AEA, also known as anandamide) and 2-arachidonoylglycerol (2-AG) are eicosanoid molecules produced from arachidonic acid. These two lipids can activate cannabinoid receptors under various physiological circumstances; they are produced both in the brain and in peripheral tissues, and then secreted by different cell types [1,2]. Endocannabinoids are involved in many physiological and pathophysiological processes, including migraine pain [1,3,4]. In response to neuronal activity, elicited, for instance, by stress, pain, or inflammatory stimulus, 2-AG and AEA are synthesized ‘on demand’ at postsynaptic sites. They then diffuse to retrogradely activate presynaptic CB1 and other receptors, causing a transient and long-lasting reduction in mediator release, resulting in analgesia [5,6]. The activation of trigeminal CB1 receptors inhibits calcitonin gene-related peptide (CGRP) release [7] and dural vasodilation [8], which are the hallmarks of migraine pathophysiology [9]. Cannabinoids or endocannabinoid system modulators may control pain through neuro-immune interactions in glial cells, which express various endocannabinoid components [10,11]. The involvement of endocannabinoid signaling in the functional coupling of neurons, astrocytes, and microglia supports its contribution to the (neuro)inflammation process [12].

Inhibitors of enzymes involved in endocannabinoid hydrolysis may reduce migraine-related pain by modulating the endocannabinoid system [13,14], although the mechanisms of action underlying this activity are still unclear. In recent years, we have devoted much of our research to inhibiting endocannabinoid degradation in our pre-clinical migraine model based on nitroglycerin (NTG) administration, demonstrating its therapeutic potential. The NTG model is a widely used animal model of migraine that, both in the acute and chronic form, may reproduce some of the features that can be found in migraine patients. Indeed, it produces cephalic and extra-cephalic hypersensitivity, allodynia, and hyperalgesia, as well as the activation of brain areas involved in migraine pain. In addition, the NTG model may reproduce some of the associated symptoms, like photophobia, and comorbid conditions such as anxiety/depression-like behavior [15,16]. This model has also been extensively used to investigate the effects of endocannabinoid system modulators [17,18,19,20,21,22,23,24]. We have shown that under conditions of hyperalgesia, endocannabinoids play a prominent role in reducing inflammatory and pain mediators in central and peripheral regions [22,23,24].

The piperazine derivative AKU-005 (4-Benzhydrylpiperazin-1-yl) (1H-1,2,4-triazol-4-yl) methadone) has been shown in vitro to be a new potent inhibitor of monoacylglycerol lipase (MAGL) and fatty acid amide hydrolase (FAAH) enzyme activity in several areas of the nervous system involved in migraine pain signaling [25]. Specifically, AKU-005 reduced meningeal afferent excitability [25] via the activation of CB1 receptors.

Interestingly, in mouse brain membrane preparations, AKU-005 can inhibit MAGL activity at sub-nanomolar concentrations (IC50 values range from 0.2 to 1.1 nM) [26], but Della Pietra et al. recently showed in human and rat meningeal samples that the basal FAAH activity is higher than the barely perceptible MAGL activity [27]. The situation is opposite in the TGs and dorsal root ganglia where strong MAGL/low FAAH activity was reported [25,27]. All the abovementioned studies were conducted in vitro, but it is evident that, due to the redundancy and promiscuity of the enzymes involved in the degradation of endocannabinoids, and also because inhibiting these enzymes may have unpredictable effects, even concerning the dose used, it is extremely important to assess their activity in vivo.

In this study, we tested for the first time the pharmacological activity of AKU-005 in vivo in the animal model of migraine based on the administration of NTG to validate its potential effect in migraine treatment.

## 2. Materials and Methods

### 2.1. Animals and Drugs

Male Sprague Dawley rats (150–175 g, Charles River Laboratories, Calco, Italy) were housed in pairs in cages at the University of Pavia’s animal facility under carefully monitored conditions (temperature 21–22 °C, relative humidity 60–50%, and 12/12 h light cycle) with water and food ad libitum. The Italian Ministry of Health (376/2020-PR) approved all procedures, which were performed in compliance with the guidelines of European Community Directive 2010/63/EU of 22 September 2010.

NTG (Bioindustria L.I.M., Novi Ligure, Italy) was prepared and administered as previously described [23]. NTG was administered intraperitoneally (i.p.) at a dose of 10 mg/kg.

AKU-005 (synthetized by the Department of Chemistry, University of Eastern Finland) was delivered systemically (i.p.) at a dose of 0.5 mg/kg in a volume of 2 mL/kg after being dissolved in tween-80/polyethylene glycol 200/saline (10/10/80; utilized as a vehicle). Considering the lack of pharmacokinetic studies on AKU-005 in vivo, the dose was calculated empirically, based on our previous experience with a similar compound tested in the same experimental paradigm [23].

### 2.2. Experimental Plan

**Figure 1:**
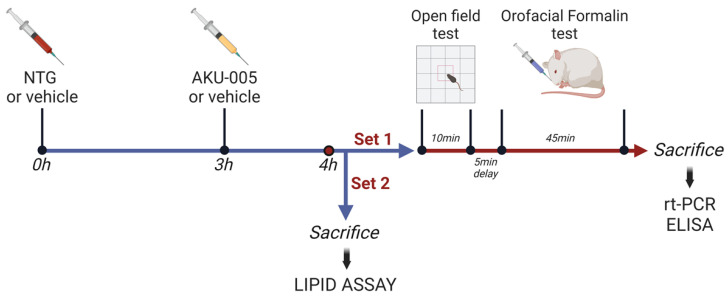
Experimental timeline for the treatment and testing procedures. After treatments, rats belonging to Set 1 underwent the open field test (10 min duration), and after 5 min of delay, they were subjected to the orofacial formalin test. At the end of behavioral testing, the animals were sacrificed and tissue/blood samples collected for gene (by rt-PCR) and protein expression (by ELISA). The animals belonging to Set 2 were immediately sacrificed after treatments, and tissue samples were collected for lipid assay (by means of mass spectrometry). Figure created with BioRender.com (https://www.biorender.com/ accessed on 7 March 2024).

Rats were randomly allocated the four experimental groups (Table 1) and used in the experimental setting reported in Figure 1.

In experimental Set 1, AKU005 (or vehicle) was administered 3 h after NTG (or its vehicle), which is 1 h before the behavioral testing. Animals underwent the open field test and, after a 5 min rest, the orofacial formalin test. At the end of the behavioral testing sessions, rats were sacrificed with a lethal dose of anesthetic (sodium thiopental, 150 mg/Kg, i.p.) followed by decapitation; truncal blood and cranial tissues were collected for ex vivo analysis.

In experimental Set 2, AKU-005 (or vehicle) was administered 3 h after NTG (or its vehicle), and after 1 h, rats were immediately sacrificed by decapitation after exposure to carbon dioxide, in order to collect the samples for lipid assays in specific brain areas.

### 2.3. Open Field Test

As many physiological and behavioral functions are influenced by endocannabinoid signaling, the open field test was used to assess anxiety, exploratory behaviors, and locomotor ability. After 60 min of acclimatization to the test room, each rat was positioned in the open field arena (Ugo Basile, Gemonio, VA, Italy) and tested for ten minutes as previously reported [24]. The room’s light was kept constant from center to corner regions of the arena, thus avoiding possible confounding results since NTG can evoke photophobia. To assess locomotor activity, anxiety, and exploration, we recorded for ten minutes the distance traveled across the arena, the time spent in the center, and rearing behaviors. Furthermore, we looked at spontaneous grooming activity as a sign of heightened nociception. An observer, blinded to the treatment, manually evaluated rearing and grooming behaviors by counting the time the animal stood on its hind legs and the time it spent face- and body-grooming, respectively. As regards the total distance and the time spent in the center, these parameters were assessed by means of the ANY-Maze software (Ugo Basile, application version 4.99g Beta) [24]. This latter was used to conventionally divide the arena into 16 square units to delineate the center of the arena, identified by the 4 central squares, and the 12 surrounding squares represent the periphery.

### 2.4. Orofacial Formalin Test

On the experimental day, the rats were injected subcutaneously with 50 μL of formalin 1.5% (made of formaldehyde 37% in water and 0.9% saline, *v*/*v*) into the right upper lip, just lateral to the nose, and positioned in an observation box. A camera, recording face-rubbing time, was located 50 cm from the box, a 30 × 30 × 30 cm glass chamber with mirrored sides, to provide a clear view of each rat. Face-rubbing was evaluated by a researcher blind to group assignment who counted the seconds the animal spent grooming the injected area with the ipsilateral forepaw or hind paw 0–3 min (Phase I) and 12–45 min (Phase II) after formalin injection. The observation time was divided into 15 blocks of 3 min each. Phase I reflects an acute pain, while Phase II represents the combined effects of afferent input and central sensitization. The orofacial formalin test was utilized in conjunction with the NTG model to induce a state of hyperalgesia that mimics the clinical condition [24].

### 2.5. Gene Expression

At the end of behavioral testing, rats belonging to Set 1 were euthanized (sodium thiopental, 150 mg/kg, i.p.).

After decapitation, meninges and medulla in toto, cervical spinal cord (CSC, C1–C2), and TG ipsilateral to formalin injection were quickly dissected out, rinsed in cold sterile 0.9% NaCl solution, placed in cryogenic tubes and immediately frozen in liquid nitrogen, and then stored at −80 °C until further processing. Tissue samples were homogenized by means of ceramic beads (PRECELLYS, Bertin Pharma, Montigny le Bretonneux—France) with TRIzol^®^ (Invitrogen, Waltham, MA, USA) to extract the total RNA. All procedures were performed under RNase-free conditions. RNA quality was assessed using a nanodrop spectrophotometer (Euroclone, Pero, MI, Italy) showing that the absorbance ratios (260/280 nm) ranged from 1.9 to 2.0 in all samples, indicating no significant protein (including of blood origin) contamination. The iScript cDNA Synthesis kit (BIO-RAD, Hercules, CA, USA) was used to generate cDNA, and the Fast Eva Green supermix (BIO-RAD, Hercules, CA, USA) was used to assess gene expression. Specifically, we evaluated the gene expression levels of *Calca* (coding for CGRP), tumor necrosis factor alpha (*TNF-alpha*), and interleukin (*IL-6*) using rt-PCR [23,24]. Glyceraldehyde 3-phosphate dehydrogenase (*GAPDH*), the expression of which remained constant in all experimental groups, was used for normalization. The following specific primers were used: *GAPDH* AACCTGCCAAGTATGATGAC (forward), GGAGTTGCTGTTGAAGTCA (reverse); *Calca* (coding for CGRP) CAGTCTCAGCTCCAAGTCATC (forward), TTCCAAGGTTGACCTCAAAG (reverse); *TNF-alpha* CCTCACACTCAGATCATCTTCTC (forward), CGCTTGGTGGTTTGCTAC (reverse); *IL-6* TTCTCTCCGCAAGAGACTTC (forward), and GGTCTGTTGTGGGTGGTATC (reverse). The 2^−∆∆Ct^ = 2^−(∆Ct gene−∆Ct housekeeping gene^) method was used to investigate the differences in gene expression.

### 2.6. CGRP Levels

Truncal blood, obtained from the animals belonging to Set 1, was collected in clot activator with gel separator serum tubes and centrifuged for 15 min at 1500× *g* for CGRP serum evaluations. CGRP levels were assessed using a commercial ELISA kit (α-CGRP: Elabsciences, Houston, TX, USA) and measured using a CLARIOstar microplate reader (BMG LABTECH, Ortenberg, Germany).

### 2.7. Lipids Levels

To evaluate the levels of 2-AG, AEA, palmitoylethanolamide (PEA), and oleoyl ethanolamide (OEA), the meninges, medulla in toto, CSC, and TG were collected from the animals of Set 2. Samples were weighed before homogenization in methanol with 0.8% formic acid containing cannabidiol-*d*_3_ as an internal standard. Samples were processed and analyzed as thoroughly described in a previous study [23].

Differently to the meningeal samples used for the gene expression analysis, the amount of most of the meninges used for lipid extraction were not enough so that lipids were undetectable. For this reason, the analysis of lipid levels in meningeal samples were not included in the Results section.

A 3200 QTRAP^®^ triple quadrupole mass spectrometer (Applied Biosystems Sciex, Darmstadt, Germany), coupled to an ExionLC 100 integrated high-performance liquid chromatography (HPLC) system (Applied Biosystems Sciex, Darmstadt, Germany), was used for the analysis. The chromatographic column was a monolithic C18 column (Onyx, 100 mm × 3 mm i.d., Phenomenex, Bologna, Italy) maintained at 25 °C. The gradient elution mobile phases were A (water/methanol 98:2 *v*/*v* containing 10 mM ammonium formate and 0.1% formic acid), B (methanol/acetonitrile/isopropanol 80:10:10 *v*/*v* containing 8 mM ammonium formate and 0.08% formic acid), and C (methanol).

### 2.8. Statistical Analysis

The face-grooming in Phase II of the orofacial formalin test of a previous study [24] was used to calculate the sample size need for this study. We used GPower (version 3.1.9.4) to perform an a priori power analysis, yielding a statistical power of 0.80 at an alpha level of 0.05. We supposed a difference in the total face-rubbing time between rats injected with NTG (mean 110 ± 39) and those with NTG + AKU-005 of approximately 70 s (mean 40 ± 13). We estimated a sample size of 6 rats in each experimental group with an effect size of 1.95. Because of an intergroup variability, we used a maximum of 9 rats per group. We tested all data for normality using the Kolmogorov–Smirnov normality test. We compared differences among groups using one-way ANOVA followed by post hoc Tukey’s multiple comparisons test. A *p* < 0.05 was considered statistically significant. We expressed all data as mean ± SEM and used GraphPad Prism software (version 9.3.1) to perform all statistical analyses.

## 3. Results

### 3.1. AKU-005 Effects on the NTG-Induced Behaviors

**Figure 2:**
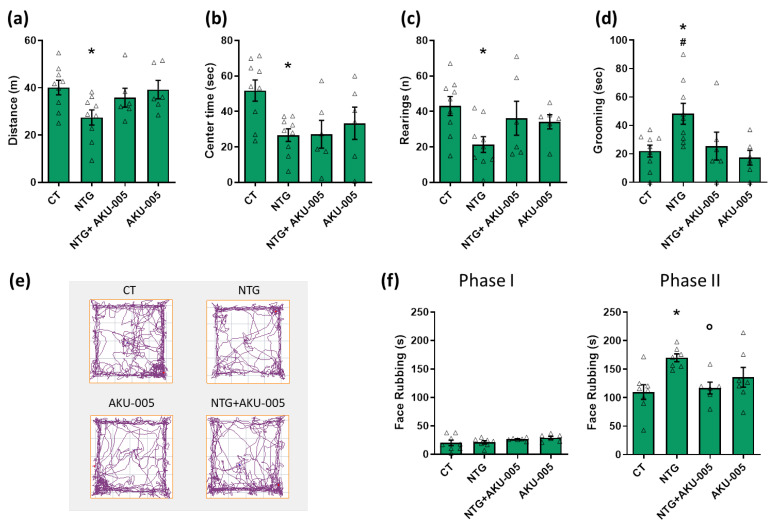
Behavioral testing. Open field test analysis: (**a**) distance (expressed in meters) travelled in the apparatus; (**b**) time spent (expressed in seconds) in the center of the apparatus; (**c**) number of rearings; (**d**) time spent performing grooming behavior (expressed in seconds); (**e**) representative track plots of the experimental groups; and (**f**) face-rubbing behavior (expressed in seconds) of Phase I and II of the orofacial formalin test. Data are expressed as mean ± SEM. One-way ANOVA followed by Tukey’s multiple comparisons test; * *p* < 0.05 vs. CT; # *p* < 0.05 vs. AKU-005; ° *p* < 0.05 vs. NTG. Individual subjects’ values are represented as triangle; N = 6–9.

The systemic administration of NTG reduced locomotor activity and exploratory behavior, expressed as distance traveled and number of rearings, compared with the CT group (Figure 2a,c). It also increased anxiety-like behavior, as indicated by the reduced time spent in the center of the open field, and nociception, as suggested by the increased grooming behavior, compared to the CT group (Figure 2b,d). AKU-005 did not alter the locomotor activity or any other behavior when injected alone, nor it affect the changes induced by NTG administration (Figure 2a–d).

As regards the orofacial formalin test, AKU-005 administration significantly prevented the NTG-induced increase in face-rubbing behavior in phase II of the test (Figure 2f). When AKU-005 was administered alone, without NTG, it did not affect the behavioral response in the test (Figure 2f).

The data show that AKU-005 can counteract the NTG-induced hyperalgesia but does not influence other migraine-like features induced by the NTG challenge.

### 3.2. AKU-005 Effects on CGRP Expression and Serum Levels

**Figure 3:**
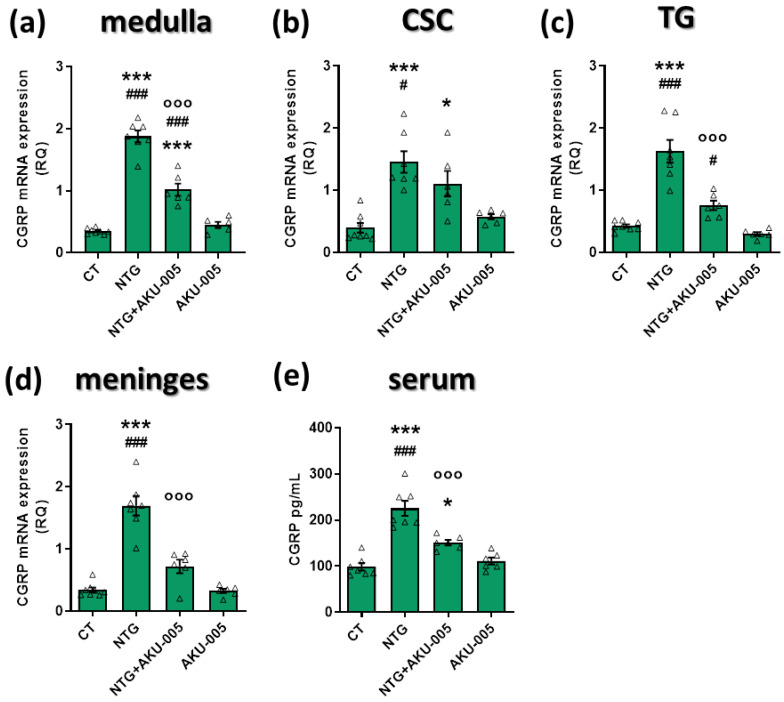
Gene expression and serum protein levels of CGRP. mRNA expression levels of *CGRP* in the (**a**) medulla, (**b**) cervical spinal cord (CSC), (**c**) trigeminal ganglion (TG), and (**d**) meninges; (**e**) CGRP serum levels (pg/mL). Data are expressed as mean ± SEM. One-way ANOVA followed by Tukey’s multiple comparisons test; * *p* < 0.05 and *** *p* < 0.001 vs. CT; °°° *p* < 0.001 vs. NTG; # *p* < 0.05 and ### *p* < 0.001 vs. AKU-005. Individual subjects’ values are represented as triangle; N = 6–8.

The administration of NTG treatment increased *CGRP* mRNA levels in the meninges, medulla, CSC, and TG ipsilateral to formalin injection as well as CGRP serum levels compared to the CT group (Figure 3a–e). AKU-005 administration significantly attenuated these changes (Figure 3a–e), except for *CGRP* in the CSC (Figure 3b). These findings suggest that the anti-hyperalgesic effects of AKU-005 are associated with a reduction in the CGRP-related pathways, contributing to pain reduction.

### 3.3. AKU-005 Effects on the Gene Expression of Pro-Inflammatory Cytokines

**Figure 4:**
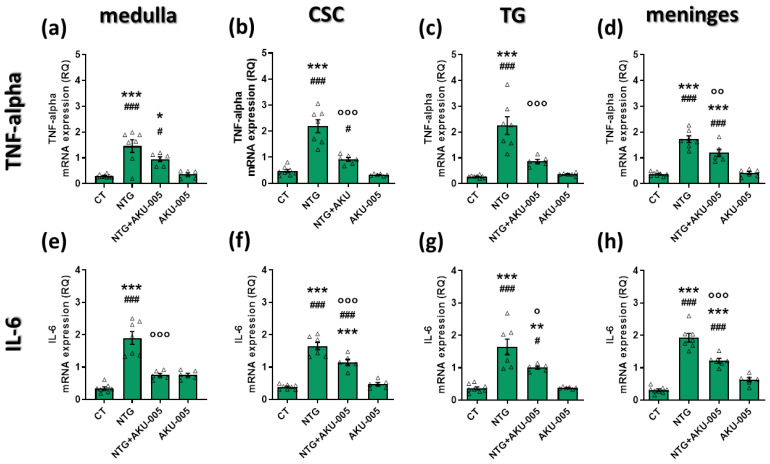
mRNA expression levels of (**a**–**d**) *TNF-alpha* and (**e**–**h**) *IL-6* in the medulla, cervical spinal cord (CSC), trigeminal ganglion (TG), and meninges. Data are expressed as mean ± SEM. One-way ANOVA followed by Tukey’s multiple comparisons test; * *p* < 0.05, ** *p* < 0.01 and *** *p* < 0.001 vs. CT; ° *p* < 0.05, °° *p* < 0.01 and °°° *p* < 0.001 vs. NTG; # *p* < 0.05 and ### *p* < 0.001 vs. AKU-005. Individual subjects’ values are represented as triangle; N = 6–8.

*TNF-alpha* and *IL-6* gene expression was significantly higher in the medulla, CSC, and TG ipsilateral to formalin injection in NTG-treated rats. NTG administration also increased the gene expression of cytokines at the meningeal level (Figure 4a–h). AKU-005 treatment prevented an NTG-induced increase in *IL-6* and *TNF-alpha* mRNA levels in all areas, except for *TNF-alpha* in the medulla (Figure 4a).

Overall, the data suggest that AKU-005 can counteract the NTG-induced activation of the inflammatory pathway in both peripheral and central areas relevant to migraine pathophysiology.

### 3.4. AKU-005 Effects on Cranial Lipid Levels

**Figure 5:**
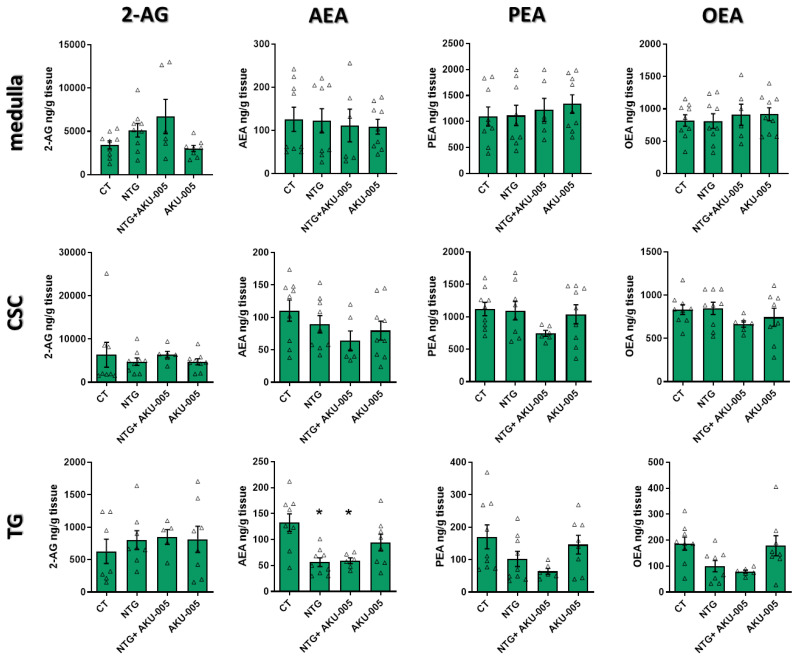
Levels of endocannabinoids and related lipids (expressed as ng/g tissue) in the medulla, cervical spinal cord (CSC), and trigeminal ganglion (TG). Data are expressed as mean ± SEM. One-way ANOVA followed by Tukey’s multiple comparisons test; * *p* < 0.05 vs. CT. Individual subjects’ values are represented as triangle; N = 6–9. Legend: 2-AG, 2-arachidonoylglycerol; AEA, arachidonoylethanolamine; PEA, palmitoylethanolamide; OEA, oleoyl ethanolamide.

The level of endocannabinoids and related lipids were below the detection threshold in the meninges. In the other areas/tissues, NTG administration did not alter the levels of endocannabinoids and related lipids when compare with the control group, except for a reduction in AEA levels in the TG. The administration of AKU-005 had no effect on such a change (Figure 5). It is reasonable to assume that AKU-005 does not strongly affect the modulation of endocannabinoids and related lipids in these brain regions.

## 4. Discussion

The local increase of AEA and 2-AG levels synthetized on demand using specific catabolic enzyme inhibitors may represent a means to modulate the endocannabinoid system while limiting the possible adverse side effects.

Here, we tested in vivo the biological activity of the reversible dual AKU-005 FAAH/MAGL inhibitor, which showed potential anti-migraine activity in vitro [27].

Our main findings can be summarized as follows:

(1) The administration of AKU-005 prevented multiple NTG-induced effects: hyperalgesia in the trigeminal region, and CGRP increase in serum and in cranial tissues and brain areas that are relevant to migraine pathophysiology;
(2) The administration of AKU-005 also prevented the NTG-induced activation of the inflammatory response;
(3) These activities were not associated with significant changes in the levels of endocannabinoids or related lipids in cranial tissues and brain areas that are relevant to migraine pathophysiology.

Altogether, these results, obtained in a migraine-specific animal model, suggest that the dual inhibition of FAAH/MAGL activity has a role in the pathophysiology of migraine. The lack of changes in the levels of endocannabinoids and related lipids in areas that are crucial for migraine pathophysiology came as a surprise. Indeed, several papers have demonstrated that the specific inhibition of FAAH or MAGL, the catabolic enzymes for AEA and 2-AG, respectively, counteracts the NTG-induced effects that are relevant to migraine [17,22,23,24,28]. Furthermore, the in vitro characterization study of AKU-005 by Aaltonen et al. [26] showed that the compound activity is dependent on the elevation of 2-AG and AEA brain levels, acting indirectly on the CB1 receptor. In a previous in vivo study, we also demonstrated that a dual FAAH/MAGL inhibitor counteracted NTG-induced effects via mechanisms that require the activation of CB1 receptors [24].

One possibility to explain why the biological activities of AKU-005 did not impact lipids in our experiments is that AKU-005 may have acted mainly at the level of the trigeminovascular terminals in the meninges, where we were unable to detect endocannabinoids. This may be due to the insufficient amount of meningeal samples needed for lipid extraction, as the trigeminovascular endings are located in the supratentorial dura mater [29].

Along this line of reasoning, we can speculate that AKU-005 induced changes in the lipid levels in the dura mater, which then caused indirect effects on other areas and, consequently, on the behavioral changes observed in the orofacial formalin test.

Similar to the present results, the dual inhibitor JZL195 significantly reduced NTG-induced trigeminal hyperalgesia and pain-associated behavior through cannabinoid receptor-mediated effects [24]. JZL195 also reduced *CGRP* and cytokine gene expression in central and peripheral areas and serum CGRP levels, suggesting that the two dual inhibitors share at least some mechanisms in their biological activity in the NTG paradigm [24]. In this scenario, we can speculate that NTG administration induced the degranulation of meningeal mast cells [30,31], thus contributing to the sensitization and activation of meningeal afferents [32] and causing a localized release of endocannabinoids, which went undetected in our experiments. In agreement, an over-regulation of inducible nitric oxide synthase (*iNOS*) gene expression was reported in the meningeal tissue after NTG administration, confirming an inflammatory state. The iNOS immunoreactivity was mainly expressed within resident meningeal macrophages and was associated with increased IL-1β expression [33]. In line with our data, AKU-005 caused minimal meningeal fiber firing under baseline conditions, while it significantly decreased meningeal firing following KCl stimulation [27], causing an increase in endocannabinoid release. The increase in endocannabinoid levels induced by AKU-005 was mediated by the CB1 receptor [25,27]. In agreement, the enhanced AEA signaling after FAAH inhibition produced significant anti-nociceptive effects in the meninges [34]. In addition, FAAH inhibition was reported to modulate pro-inflammatory activity [35] by interacting with several ion channels expressed in BV2 microglial cells, such as the potassium channel Kv1.5 [36] or the L-type calcium channel [37]. Other endocannabinoid catabolic enzyme inhibitors have shown different effects depending on the dose or timing of administration [38,39]. In addition, it is possible that AKU-005 may had an off-target effect [40,41] which has not yet been identified. For instance, PF3845 and URB597 were able to reduce prostaglandin E_2_ (PGE2) production in lipopolysaccharide-stimulated BV2 microglial cells by suppressing the expression of PGE2 biosynthesis rather than the blockade of PGE2 biosynthetic enzymes [42]. It cannot be ruled out that AKU-005, with the dose utilized in vivo, may cause changes in other metabolites that are not closely related to the ECs in the areas investigated [43], thus causing a reduction in nociception through the modulation of CGRP release.

Of course, additional studies on the molecular and cellular characterization of AKU-005 and, specifically, on the assessment of its functional profile in the meninges and/or other brain areas are necessary to further elaborate on this hypothesis; but, still, we feel that the present findings suggest the opportunity to further investigate the high therapeutic potential of this pathway based on the dual inhibition of MAGL and FAAH activity.

### Limitations of the Study

Although our animal model is commonly used for studying migraine [15,16], it has some limitations, like any other model. In the NTG human model, only individuals predisposed to migraine are subjected to develop NTG-induced headache, with varying onset times [15,16,44]. Using the animal model, we can mimic only a few aspects of the disease, such as the change in pain sensitivity induced by NTG through the action of CGRP release. In this context, AKU-005 seems to be an ideal candidate as it reduces nociception generation by modulating CGRP release. However, this was a single-dose study, with a dose selected pragmatically due to the lack of informative evidence on AKU-005 in vivo effects. Thus, additional studies are necessary to assess whether the lack of effects of AKU-005 on some of the parameters assessed may be related to an insufficient dose or to tissue-specific mechanisms. The latter seem particularly relevant in pain processing [45]. In this context, a thorough pharmacokinetic profile of the compound and a dose–response assessment will provide crucial information for further studies. Furthermore, we sampled lipid levels only once at a given time point. More information can be derived from a time-course evaluation and from an optimized methodology for obtaining adequate specimens of the meninges.

## 5. Conclusions

The present findings show for the first time the in vivo potential of the dual MAGL/FAAH inhibitor AKU-005 to counteract several of the NTG-induced effects that are relevant to migraine, probably by modulating the CGRP pathway. Further assessment of the pathways and mediators involved in this biological activity will inform the pathophysiological mechanism of migraine and possibly further characterize a pathway with a high druggable potential.

## Figures and Tables

**Table 1:** Experimental groups. Each animal received two i.p. injections, indicated as Treatment 1 and 2.

Group Name	Treatment 1	Treatment 2
Control (CT)	NTG vehicle	AKU-005 vehicle
NTG	NTG 10 mg/kg	AKU-005 vehicle
NTG + AKU-005	NTG 10 mg/kg	AKU-005 0.5 mg/kg
AKU-005	NTG vehicle	AKU-005 0.5 mg/kg

## Data Availability

The data presented in this study are available in Zenodo repository [doi:10.5281/zenodo.10817751].

## References

[1] Piomelli D. (2005). The Endocannabinoid System: A Drug Discovery Perspective. Curr. Opin. Investig. Drugs.

[2] Finn D.P., Haroutounian S., Hohmann A.G., Krane E., Soliman N., Rice A.S.C. (2021). Cannabinoids, the Endocannabinoid System, and Pain: A Review of Preclinical Studies. Pain.

[3] Lo Castro F., Baraldi C., Pellesi L., Guerzoni S. (2022). Clinical Evidence of Cannabinoids in Migraine: A Narrative Review. J. Clin. Med..

[4] Akerman S., Holland P.R., Lasalandra M.P., Goadsby P.J. (2013). Endocannabinoids in the Brainstem Modulate Dural Trigeminovascular Nociceptive Traffic via CB _1_ and “Triptan” Receptors: Implications in Migraine. J. Neurosci..

[5] Kano M., Ohno-Shosaku T., Hashimotodani Y., Uchigashima M., Watanabe M. (2009). Endocannabinoid-Mediated Control of Synaptic Transmission. Physiol. Rev..

[6] Di Marzo V., Bisogno T., De Petrocellis L. (2007). Endocannabinoids and Related Compounds: Walking Back and Forth between Plant Natural Products and Animal Physiology. Chem. Biol..

[7] Fischer M., Messlinger K. (2007). Cannabinoid and Vanilloid Effects of R(+)-Methanandamide in the Hemisected Meningeal Preparation. Cephalalgia.

[8] Akerman S., Kaube H., Goadsby P.J. (2004). Anandamide Acts as a Vasodilator of Dural Blood Vessels In Vivo by Activating TRPV1 Receptors. Br. J. Pharmacol..

[9] Goadsby P.J., Holland P.R., Martins-Oliveira M., Hoffmann J., Schankin C., Akerman S. (2017). Pathophysiology of Migraine: A Disorder of Sensory Processing. Physiol. Rev..

[10] Luongo L., Maione S., Di Marzo V. (2014). Endocannabinoids and Neuropathic Pain: Focus on Neuron–Glia and Endocannabinoid–Neurotrophin Interactions. Eur. J. Neurosci..

[11] Cabañero D., Ramírez-López A., Drews E., Schmöle A., Otte D.M., Wawrzczak-Bargiela A., Huerga Encabo H., Kummer S., Ferrer-Montiel A., Przewlocki R. (2020). Protective Role of Neuronal and Lymphoid Cannabinoid CB2 Receptors in Neuropathic Pain. Elife.

[12] Kasatkina L.A., Rittchen S., Sturm E.M. (2021). Neuroprotective and Immunomodulatory Action of the Endocannabinoid System under Neuroinflammation. Int. J. Mol. Sci..

[13] Leimuranta P., Khiroug L., Giniatullin R. (2018). Emerging Role of (Endo)Cannabinoids in Migraine. Front. Pharmacol..

[14] Liktor-Busa E., Levine A.A., Palomino S.M., Singh S., Wahl J., Vanderah T.W., Stella N., Largent-Milnes T.M. (2023). ABHD6 and MAGL Control 2-AG Levels in the PAG and Allodynia in a CSD-Induced Periorbital Model of Headache. Front. Pain Res..

[15] Demartini C., Greco R., Zanaboni A.M., Sances G., De Icco R., Borsook D., Tassorelli C. (2019). Nitroglycerin as a Comparative Experimental Model of Migraine Pain: From Animal to Human and Back. Prog. Neurobiol..

[16] Sureda-Gibert P., Romero-Reyes M., Akerman S. (2022). Nitroglycerin as a Model of Migraine: Clinical and Preclinical Review. Neurobiol. Pain.

[17] Nozaki C., Markert A., Zimmer A. (2015). Inhibition of FAAH Reduces Nitroglycerin-Induced Migraine-like Pain and Trigeminal Neuronal Hyperactivity in Mice. Eur. Neuropsychopharmacol..

[18] Nagy-Grócz G., Bohár Z., Fejes-Szabó A., Laborc K.F., Spekker E., Tar L., Vécsei L., Párdutz Á. (2017). Nitroglycerin Increases Serotonin Transporter Expression in Rat Spinal Cord but Anandamide Modulated This Effect. J. Chem. Neuroanat..

[19] Nagy-Grócz G., Tar L., Bohár Z., Fejes-Szabó A., Laborc K.F., Spekker E., Vécsei L., Párdutz Á. (2016). The Modulatory Effect of Anandamide on Nitroglycerin-Induced Sensitization in the Trigeminal System of the Rat. Cephalalgia.

[20] Peng L.-M., Chen X.-P., Shi R.-Z., Chen L., Li Y.-J., Yang T.-L. (2014). Involvement of Anandamide Transporter in Calcitonin Gene-Related Peptide Expression Stimulated by Nitroglycerin and Influence of ALDH2 Glu504Lys Polymorphism. J. Cardiovasc. Pharmacol..

[21] Mansoori M., Zarei M.R., Chamani G., Nazeri M., Mohammadi F., Alavi S.S., Shabani M. (2020). Chronic Migraine Caused a Higher Rate of Tendency to Cannabinoid Agonist Compared to Morphine. Acta Biomed..

[22] Greco R., Demartini C., Zanaboni A., Casini I., De Icco R., Reggiani A., Misto A., Piomelli D., Tassorelli C. (2021). Characterization of the Peripheral FAAH Inhibitor, URB937, in Animal Models of Acute and Chronic Migraine. Neurobiol. Dis..

[23] Greco R., Francavilla M., Demartini C., Zanaboni A.M., Facchetti S., Palmisani M., Franco V., Tassorelli C. (2023). Activity of FAAH-Inhibitor JZP327A in an Experimental Rat Model of Migraine. Int. J. Mol. Sci..

[24] Greco R., Demartini C., Francavilla M., Zanaboni A.M., Tassorelli C. (2021). Dual Inhibition of FAAH and MAGL Counteracts Migraine-like Pain and Behavior in an Animal Model of Migraine. Cells.

[25] Della Pietra A., Giniatullin R., Savinainen J.R. (2021). Distinct Activity of Endocannabinoid-Hydrolyzing Enzymes MAGL and FAAH in Key Regions of Peripheral and Central Nervous System Implicated in Migraine. Int. J. Mol. Sci..

[26] Aaltonen N., Savinainen J.R., Ribas C.R., Rönkkö J., Kuusisto A., Korhonen J., Navia-Paldanius D., Häyrinen J., Takabe P., Käsnänen H. (2013). Piperazine and Piperidine Triazole Ureas as Ultrapotent and Highly Selective Inhibitors of Monoacylglycerol Lipase. Chem. Biol..

[27] Della Pietra A., Krivoshein G., Ivanov K., Giniatullina R., Jyrkkänen H.-K., Leinonen V., Lehtonen M., van den Maagdenberg A.M.J.M., Savinainen J., Giniatullin R. (2023). Potent Dual MAGL/FAAH Inhibitor AKU-005 Engages Endocannabinoids to Diminish Meningeal Nociception Implicated in Migraine Pain. J. Headache Pain..

[28] Greco R., Demartini C., Zanaboni A.M., Berliocchi L., Piomelli D., Tassorelli C. (2018). Inhibition of Monoacylglycerol Lipase: Another Signalling Pathway for Potential Therapeutic Targets in Migraine?. Cephalalgia.

[29] Terrier L., Hadjikhani N., Velut S., Magnain C., Amelot A., Bernard F., Zöllei L., Destrieux C. (2021). The Trigeminal System: The Meningovascular Complex—A Review. J. Anat..

[30] Ghosh S., Kinsey S.G., Liu Q., Hruba L., McMahon L.R., Grim T.W., Merritt C.R., Wise L.E., Abdullah R.A., Selley D.E. (2015). Full Fatty Acid Amide Hydrolase Inhibition Combined with Partial Monoacylglycerol Lipase Inhibition: Augmented and Sustained Antinociceptive Effects with Reduced Cannabimimetic Side Effects in Mice. J. Pharmacol. Exp. Ther..

[31] Cabral G.A., Marciano-Cabral F. (2005). Cannabinoid Receptors in Microglia of the Central Nervous System: Immune Functional Relevance. J. Leukoc. Biol..

[32] Ferrari L.F., Levine J.D., Green P.G. (2016). Mechanisms Mediating Nitroglycerin-Induced Delayed-Onset Hyperalgesia in the Rat. Neuroscience.

[33] Reuter U., Bolay H., Jansen-Olesen I., Chiarugi A., Del Rio M.S., Letourneau R., Theoharides T.C., Waeber C., Moskowitz M.A. (2001). Delayed Inflammation in Rat Meninges: Implications for Migraine Pathophysiology. Brain.

[34] Cruz S.L., Sánchez-Miranda E., Castillo-Arellano J.I., Cervantes-Villagrana R.D., Ibarra-Sánchez A., González-Espinosa C. (2018). Anandamide Inhibits FcεRI-Dependent Degranulation and Cytokine Synthesis in Mast Cells through CB2 and GPR55 Receptor Activation. Possible Involvement of CB2-GPR55 Heteromers. Int. Immunopharmacol..

[35] Espinosa-Parrilla J.F., Martínez-Moreno M., Gasull X., Mahy N., Rodríguez M.J. (2015). The L-Type Voltage-Gated Calcium Channel Modulates Microglial pro-Inflammatory Activity. Mol. Cell. Neurosci..

[36] Moreno-Galindo E.G., Barrio-Echavarría G.F., Vásquez J.C., Decher N., Sachse F.B., Tristani-Firouzi M., Sánchez-Chapula J.A., Navarro-Polanco R.A. (2010). Molecular Basis for a High-Potency Open-Channel Block of Kv1.5 Channel by the Endocannabinoid Anandamide. Mol. Pharmacol..

[37] Vignali M., Benfenati V., Caprini M., Anderova M., Nobile M., Ferroni S. (2009). The Endocannabinoid Anandamide Inhibits Potassium Conductance in Rat Cortical Astrocytes. Glia.

[38] Compton D.R., Martin B.R. (1997). The Effect of the Enzyme Inhibitor Phenylmethylsulfonyl Fluoride on the Pharmacological Effect of Anandamide in the Mouse Model of Cannabimimetic Activity. J. Pharmacol. Exp. Ther..

[39] Okine B.N., Norris L.M., Woodhams S., Burston J., Patel A., Alexander S.P., Barrett D.A., Kendall D.A., Bennett A.J., Chapman V. (2012). Lack of Effect of Chronic Pre-treatment with the FAAH Inhibitor URB597 on Inflammatory Pain Behaviour: Evidence for Plastic Changes in the Endocannabinoid System. Br. J. Pharmacol..

[40] Liu J., Fike K.R., Dapper C., Klemba M. (2024). Metabolism of Host Lysophosphatidylcholine in *Plasmodium Falciparum*–Infected Erythrocytes. Proc. Natl. Acad. Sci. USA.

[41] Bosier B., Muccioli G.G., Lambert D.M. (2013). The FAAH Inhibitor URB597 Efficiently Reduces Tyrosine Hydroxylase Expression through CB_1_- and FAAH-independent Mechanisms. Br. J. Pharmacol..

[42] Tanaka M., Yagyu K., Sackett S., Zhang Y. (2019). Anti-Inflammatory Effects by Pharmacological Inhibition or Knockdown of Fatty Acid Amide Hydrolase in BV2 Microglial Cells. Cells.

[43] Starowicz K., Makuch W., Korostynski M., Malek N., Slezak M., Zychowska M., Petrosino S., De Petrocellis L., Cristino L., Przewlocka B. (2013). Full Inhibition of Spinal FAAH Leads to TRPV1-Mediated Analgesic Effects in Neuropathic Rats and Possible Lipoxygenase-Mediated Remodeling of Anandamide Metabolism. PLoS ONE.

[44] Pellesi L. (2023). The Human NTG Model of Migraine in Drug Discovery and Development. Expert. Opin. Drug Discov..

[45] Rouzer C.A., Marnett L.J. (2011). Endocannabinoid Oxygenation by Cyclooxygenases, Lipoxygenases, and Cytochromes P450: Cross-Talk between the Eicosanoid and Endocannabinoid Signaling Pathways. Chem. Rev..

